# Supplementary material for: Electronic correlations and flattened band in magnetic Weyl semimetal candidate Co3Sn2S2
Source: Nat Commun. 2020 Aug 10;11:3985. doi: 10.1038/s41467-020-17234-0 (PMC7417588; doi:10.1038/s41467-020-17234-0)
Supplement: Supplementary file 1 — Supplementary Information [file 41467_2020_17234_MOESM1_ESM.pdf]

Supplementary Information for "Electronic correlations and flattened band in magnetic Weyl semimetal candidate $Co_3Sn_2S_2$"

Yueshan Xu,[1,2] Jianzhou Zhao,[3,4] Changjiang Yi,[1,2] Qi Wang,[5] Qiangwei Yin,[5] Yilin Wang,[6] Xiaolei Hu,[1,2] Luyang Wang,[7] Enke Liu,[1,8] Gang Xu,[9] Ling Lu,[1,8] Alexey A. Soluyanov,[4] Hechang Lei,[5] Youguo Shi,[1,8] Jianlin Luo,[1,8] and Zhi-Guo Chen[1,8]

[1] *Beijing National Laboratory for Condensed Matter Physics, Institute of Physics, Chinese Academy of Sciences, Beijing 100190, China*
[2] *School of Physical Sciences, University of Chinese Academy of Sciences, Beijing 100190, China*
[3] *Co-Innovation Center for New Energetic Materials, Southwest University of Science and Technology, Mianyang, Sichuan 621010, China*
[4] *Physik-Institut, Universität Zürich, Winterthurerstrasse 190, CH-8057 Zurich, Switzerland*
[5] *Department of Physics and Beijing Key Laboratory of Opto-electronic Functional Materials and Micro-nano Devices, Renmin University of China, Beijing 100872, China*
[6] *Department of Condensed Matter Physics and Materials Science, Brookhaven National Laboratory, Upton, New York 11973, USA*
[7] *Sate Key Laboratory of Optoelectronic Materials and Technologies, School of Physics, Sun Yat-Sen University, Guangzhou 510275, China*
[8] *Songshan Lake Materials Laboratory, Dongguan, Guangdong 523808, China*
[9] *Wuhan National High Magnetic Field Center, Huazhong University of Science and Technology, Wuhan, Hubei 430074, China*

Supplementary Figures

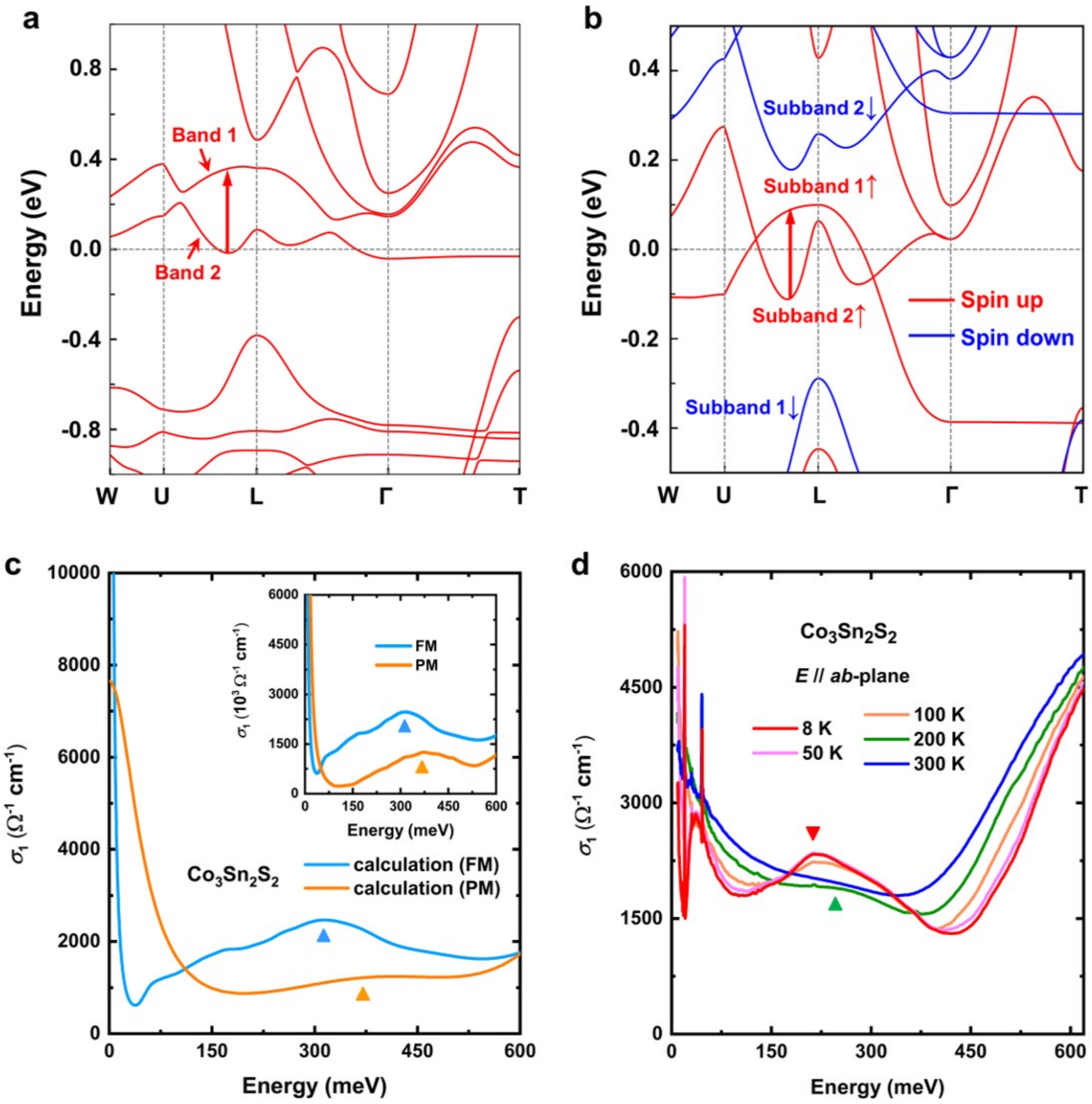

Supplementary Figure 1. Electronic structures and optical conductivity spectra in the paramagnetic (PM) and ferromagnetic (FM) states of $Co_3Sn_2S_2$. a, Spin-degenerate bands of PM $Co_3Sn_2S_2$ calculated with spin-orbit coupling (SOC). The red arrow shows the optical transition between the occupied state in Band 2 and the empty state in Band 1. **b,** Fully spin-polarized bands of FM $Co_3Sn_2S_2$ calculated without SOC. The red arrow shows the optical transition between the occupied state in Subband 2↑ and the empty state in Subband 1↑. **c,** Theoretical $\sigma_1^{\mathrm{T}}(\omega)$ of FM and PM $Co_3Sn_2S_2$ calculated with SOC. The theoretical $\sigma_1^{\mathrm{T}}(\omega)$ of FM $Co_3Sn_2S_2$ in **c** was calculated with the scattering rate of 3.5 meV. The theoretical $\sigma_1^{\mathrm{T}}(\omega)$ of PM $Co_3Sn_2S_2$ in **c** and the inset of **c** were calculated with the scattering rates of 50 meV and 5 meV, respectively. **d,** Experimental $\sigma_1^{\mathrm{E}}(\omega)$ of $Co_3Sn_2S_2$ at different temperatures.

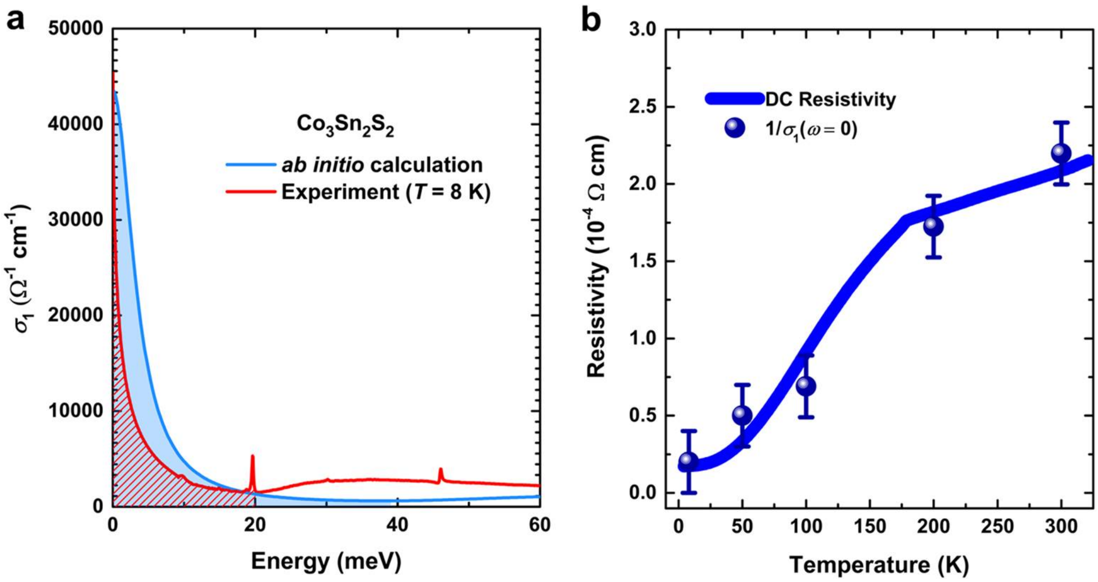

Supplementary Figure 2. Drude components of the experimental and calculated optical conductivity spectra of ferromagnetic $Co_3Sn_2S_2$ and comparison of the reciprocal value of the $\sigma_1(\omega = 0)$ with the direct current resistivity of this material. a, Drude components obtained by the optical experiment at temperature $T = 8$ K and *ab initio* calculations over a broad range of the $\sigma_1(\omega)$. The theoretical Drude component here was calculated with the scattering rate of 3.5 meV. **b,** Direct current resistivity and reciprocal value of the real part of the optical conductivity $\sigma_1(\omega = 0)$ of $Co_3Sn_2S_2$ single crystals at different temperatures.

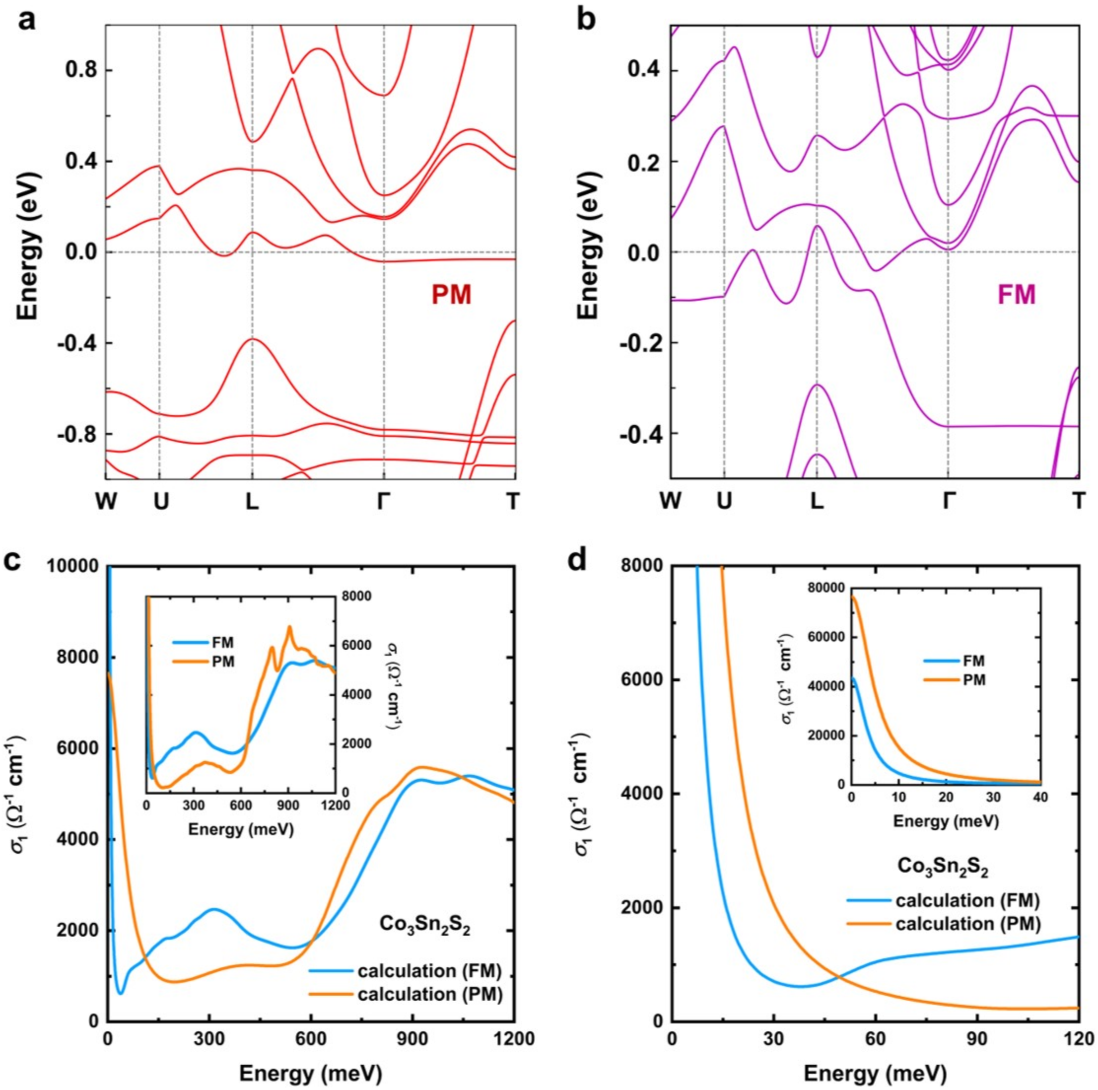

Supplementary Figure 3. Comparison of the Drude components of the calculated optical conductivity spectra in ferromagnetic (FM) and paramagnetic (PM) $Co_3Sn_2S_2$. **a,** Electronic band structure of PM $Co_3Sn_2S_2$ obtained by the density-functional-theory calculations at temperature $T = 0$ K with spin-orbit coupling. **b,** Electronic band structure of FM $Co_3Sn_2S_2$ obtained by the density-functional-theory calculations at $T = 0$ K with spin-orbit coupling. **c,** Theoretical $\sigma_1^{\mathrm{T}}(\omega)$ of FM and PM $Co_3Sn_2S_2$ calculated with SOC. The theoretical $\sigma_1^{\mathrm{T}}(\omega)$ of PM $Co_3Sn_2S_2$ in **c** and the inset of **c** were calculated with the scattering rates of 50 meV and 5 meV, respectively. The theoretical $\sigma_1^{\mathrm{T}}(\omega)$ of FM $Co_3Sn_2S_2$ in **c** and the inset of **c** was calculated with the scattering rates of 3.5 meV. **d,** Low-energy parts of the $\sigma_1^{\mathrm{T}}(\omega)$ of FM and PM $Co_3Sn_2S_2$. The inset of (d) displays the magnified view of the $\sigma_1^{\mathrm{T}}(\omega)$ of FM and PM $Co_3Sn_2S_2$. In **d**, the $\sigma_1^{\mathrm{T}}(\omega)$ of PM $Co_3Sn_2S_2$ was calculated with the scattering rates of 5 meV.

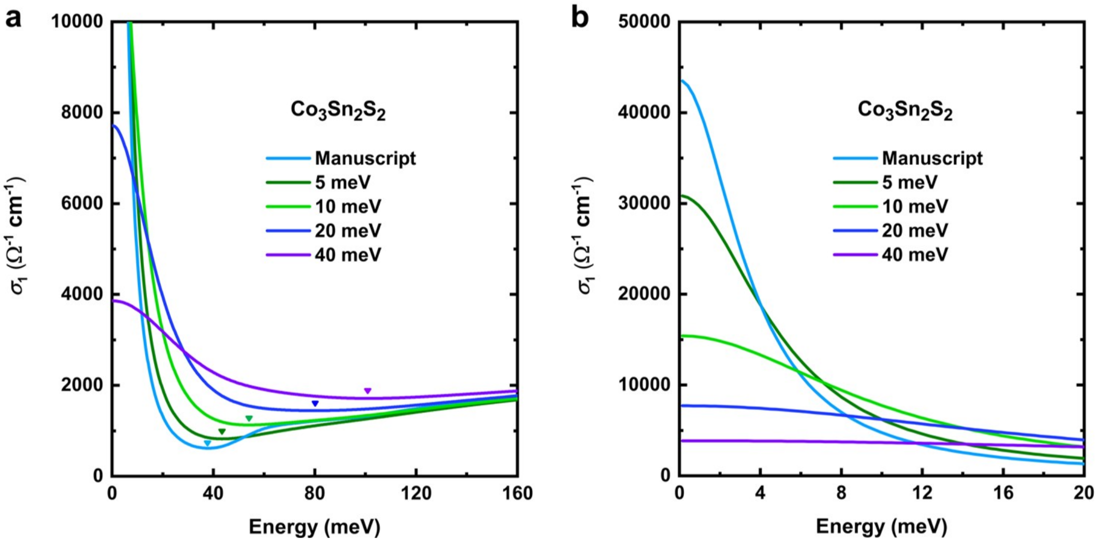

Supplementary Figure 4. Theoretical Drude components calculated using the different scattering rates in ferromagnetic (FM) $\mathbf{Co_3Sn_2S_2}$. a, Theoretical $\sigma_1^{\mathrm{T}}(\omega)$ of ferromagnetic $Co_3Sn_2S_2$ calculated using the different scattering rates $\Gamma = 3.5$ meV in the manuscript, 5 meV, 10 meV, 20 meV and 40 meV. The colored triangles indicate the chosen cutoff frequencies ($\omega_c = 38.9$ meV in the manuscript, 43.2 meV, 54.2 meV, 80.3 meV and 101.6 meV) in these theoretical $\sigma_1^{\mathrm{T}}(\omega)$. **b,** Low-energy parts of the theoretical $\sigma_1^{\mathrm{T}}(\omega)$ of ferromagnetic $Co_3Sn_2S_2$ calculated using the different scattering rates.

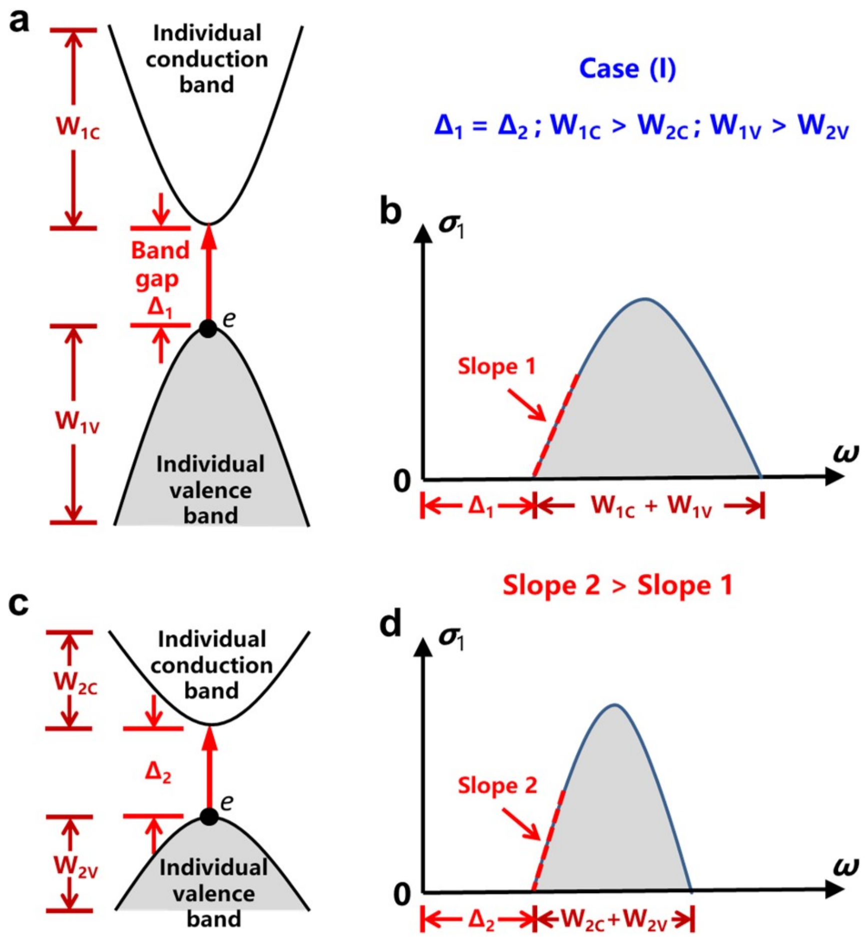

Supplementary Figure 5. Schematics of the optical interband transitions with the chemical potentials located inside the bandgaps and the corresponding peak-like features in the optical-conductivity spectra $\sigma_1(\omega)$. The energy gap Δ_1 between the conduction and valence bands in **a** is the same as the band gap Δ_2 in **c**, while the conduction-band width W_{1C} and the valence-band width W_{1V} in **a** are larger than the bandwidths W_{2C} and W_{2V} in **c**, respectively. Correspondingly, the onset energy Δ_2 of the peak-like feature in **d** is equal to that Δ_1 of the peak-like feature in **b**, while as shown by the red dashed lines in **b** and **d**, the left side of the peak in **d** is steeper than that of the peak in **b**.

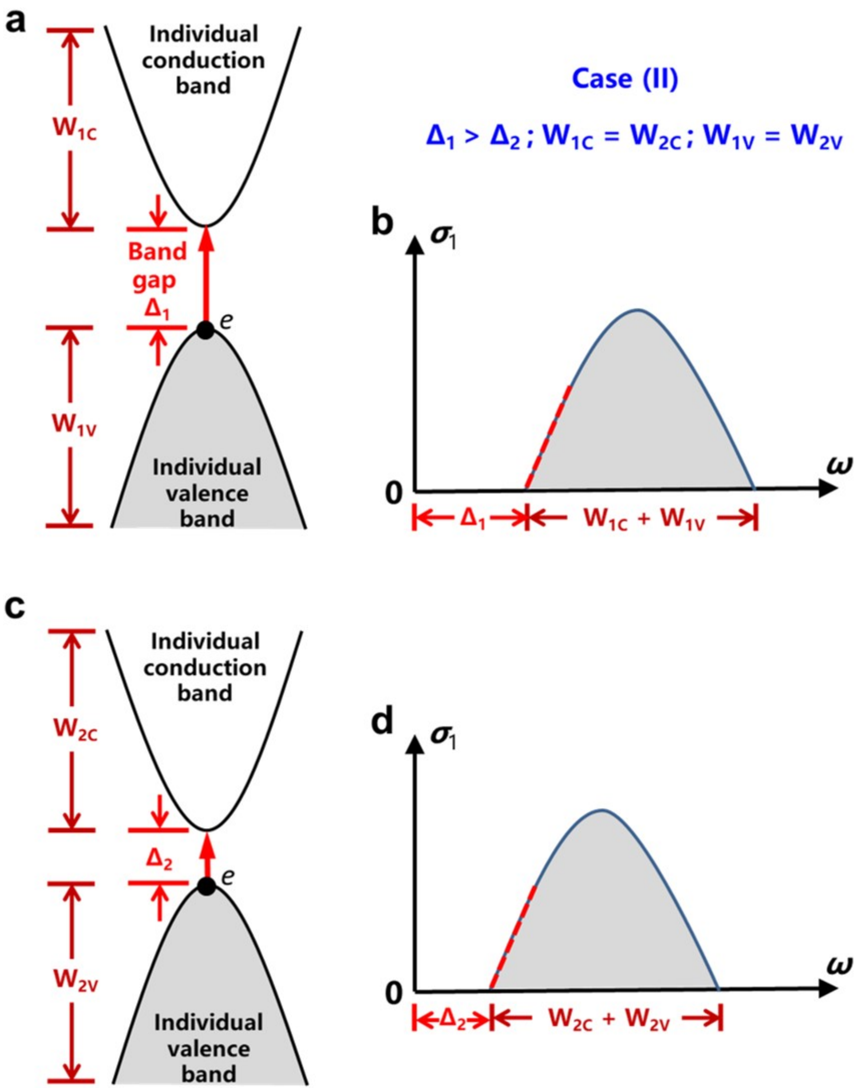

Supplementary Figure 6. Schematics of the optical interband transitions with the chemical potentials located inside the bandgaps and the corresponding peak-like features in the optical-conductivity spectra $\sigma_1(\omega)$. The energy gap Δ_1 between the conduction and valence bands in **a** is larger than the band gap Δ_2 in **c**, while the conduction-band width W_{1C} and the valence-band width W_{1V} in **a** the same as the bandwidths W_{2C} and W_{2V} in **c**, respectively. Correspondingly, the onset energy Δ_2 of the peak-like feature in **d** is smaller than the Δ_1 of the peak-like feature in **b**.

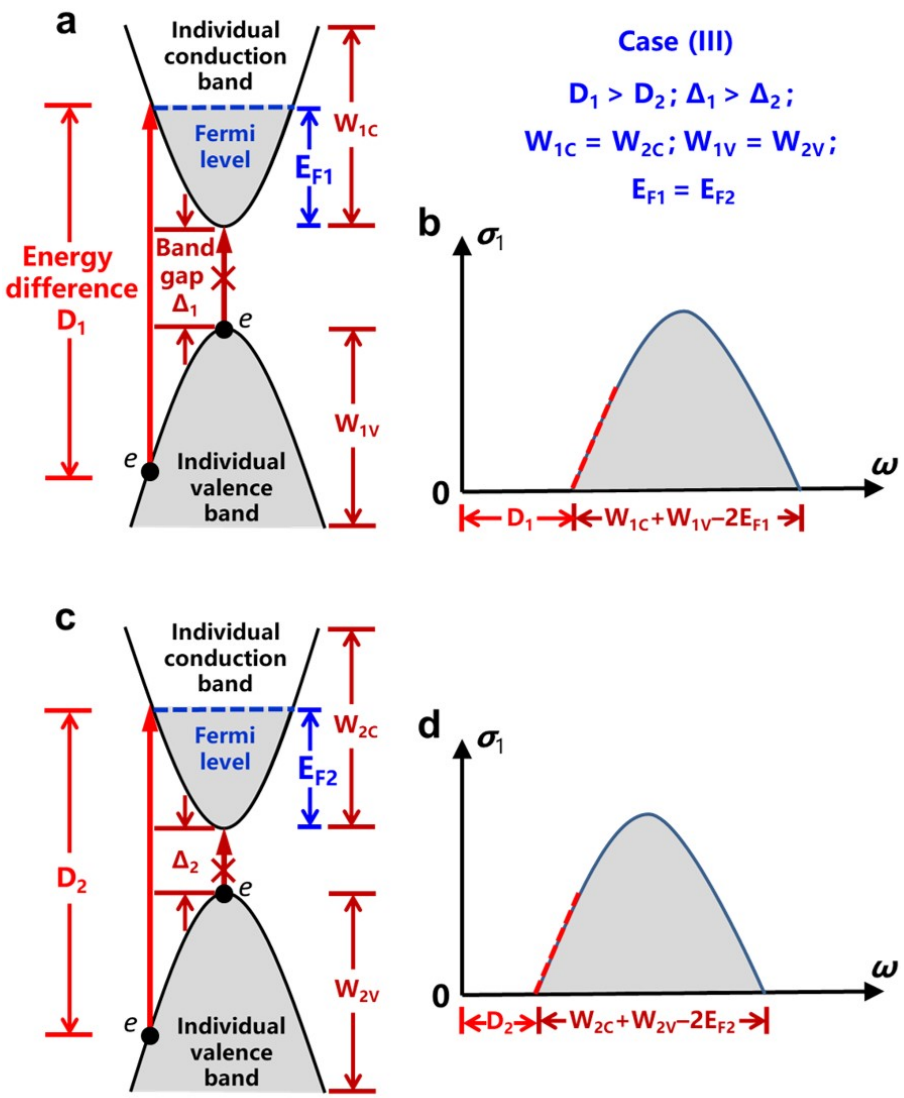

Supplementary Figure 7. Schematics of the optical interband transitions with the conduction bands partially occupied and the corresponding peak-like features in the optical-conductivity spectra $\sigma_1(\omega)$. The energy gap Δ_1 between the conduction and valence bands in **a** is larger than the band gap Δ_2 in **c**, while (i) the conduction-band width W_{1C} and the valence-band width W_{1V} in **a** the same as the bandwidths W_{2C} and W_{2V} in **c**, respectively, and (ii) the Fermi energy E_{F1} in **a** is equal to the Fermi energy E_{F2} in **c**. Correspondingly, the onset energy D_2 of the peak-like feature in **d** is smaller than the D_1 of the peak-like feature in **b**.

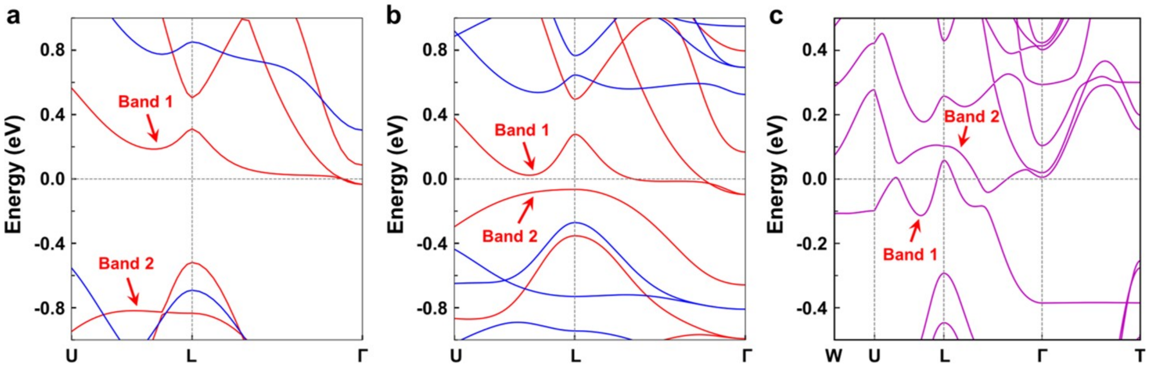

Supplementary Figure 8. Electronic band structures of ferromagnetic $Co_3Sn_2S_2$ obtained by HSE06 hybrid functional method and mBJ method. a, Band structure calculated by HSE06 hybrid functional method. **b,** Band structure calculated by mBJ method. **c,** Theoretical band structure of ferromagnetic $Co_3Sn_2S_2$ shown in our original manuscript. In **c,** the inversion between the band 1 and the band 2 occurs, while in **a** and **b**, an inversion does not exist between the band 1 and the band 2.

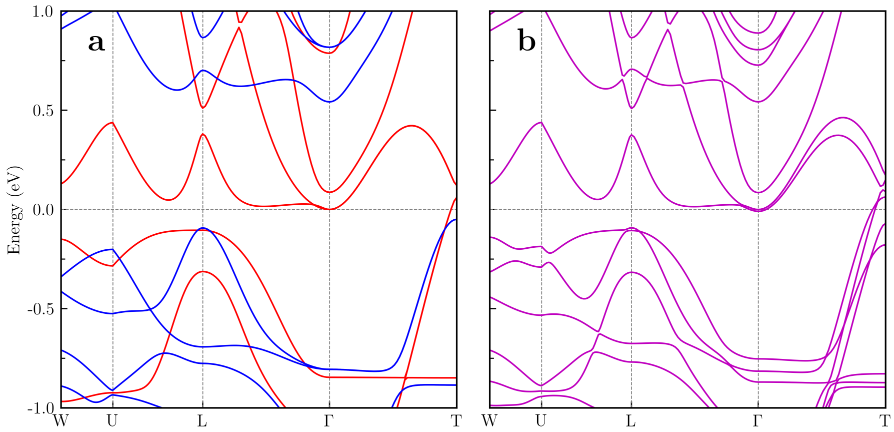

Supplementary Figure 9. Band structures obtained by generalized gradient approximation (GGA) + U calculations. a, Band structure obtained without spin-orbit coupling. **b,** Band structure obtained with spin-orbit coupling. In **a**, the spin-up and spin-down bands are colored in red and blue, respectively.

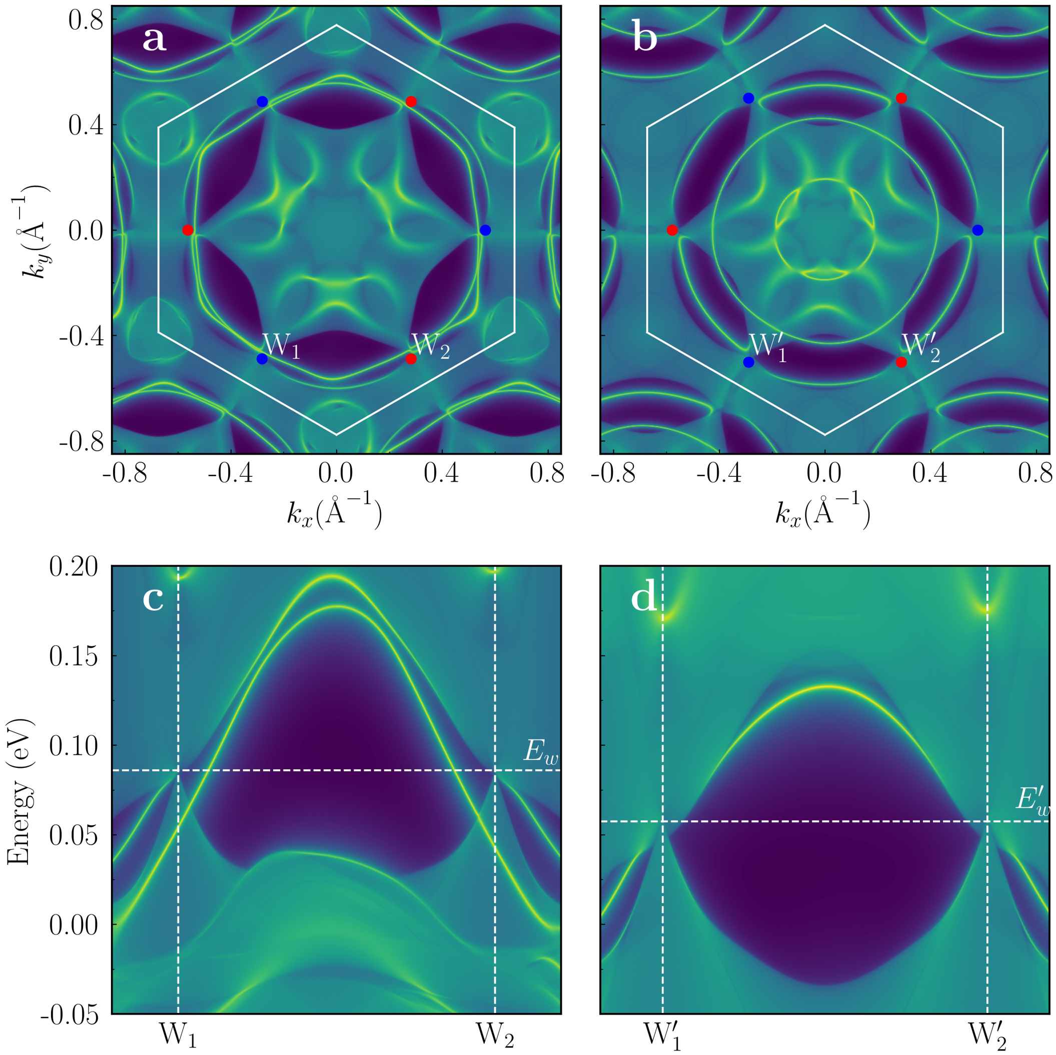

Supplementary Figure 10. Quasiparticle Fermi arcs and surface states of ferromagnetic $Co_3Sn_2S_2$. a, Quasiparticle Fermi surface on the (001) surface cutting on the energy of Weyl points E_ω in **c**. **b,** DFT Fermi surface on the (001) surface cutting on the energy of Weyl points E'_ω in **d**. **c,** Quasiparticle Energy dispersion along k path crossing one pair of Weyl points (W_1 and W_2) connected by Fermi arc. **d,** DFT Energy dispersion along k path crossing one pair of Weyl points (W'_1 and W'_2) connected by Fermi arc.

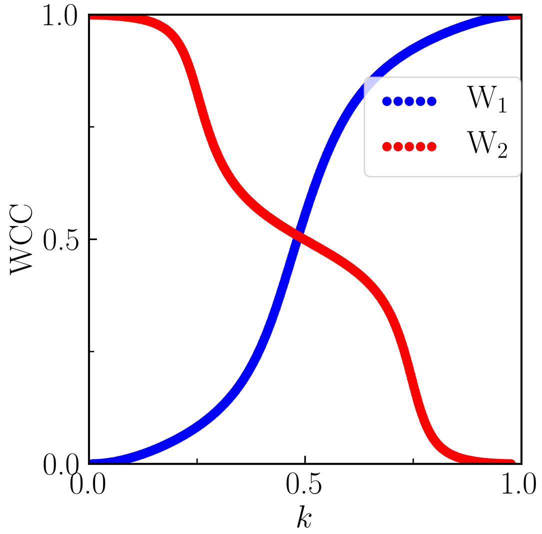

Supplementary Figure 11. Evolution of Wannier charge center (WCC) on a sphere that enclosing W_1 (in blue) and W_2 (in red) in Brillouin Zone.

Supplementary Note 1. Possible relation between the decrease in the α-peak intensity and the absence of Weyl semimetal phase

In order to find spectroscopic evidence for the absence of the magnetic Weyl semimetal phase in the paramagnetic (PM) state of $Co_3Sn_2S_2$, we not only performed single-particle ab initio calculations of its electronic band structure and the real part $\sigma_1^{\mathrm{T}}(\omega)$ of its optical conductivity in the PM state (see Supplementary Fig. 1). In comparison to Band 1 and Band 2 along the momentum path U-L-Γ near the Fermi energy in the PM state shown in Supplementary Fig. 1a here, Subband 1↑ and Subband 2↑ along the momentum path U-L-Γ near the Fermi energy in the ferromagnetic (FM) state displayed in Supplementary Fig. 1b here shift towards lower energies because across the FM transition, (i) the spin-degenerate band—Band 1 splits into the two spin- polarized subbands: Subband 1↑ and Subband 1↓ and (ii) the spin-degenerate band—Band 2 splits into the two spin-polarized subbands: Subband 2↑ and Subband 2↓. Compared with Band 2, the lowering of the energy of Subband 2↑ results in a larger occupation of Subband 2↑ (i.e., a large part of Subband 2↑ is occupied by electrons, while a minority of Band 2 is occupied), as shown in Supplementary Fig. 1a and Supplementary Fig. 1b. According to Fermi's golden rule for optical transitions, the larger occupation of Subband 2↑ implies a larger probability for optical transitions and a stronger intensity of the optical-transition-induced spectroscopic-feature. Therefore, as displayed in Supplementary Fig. 1c, the intensity of the theoretical peak-like feature around 319.7 meV in the $\sigma_1^{\mathrm{T}}(\omega)$ of FM $Co_3Sn_2S_2$, which mainly arises from the optical transitions between Subband 2↑ and Subband 1↑ (see the red arrow in Supplementary Fig. 1b) and was calculated with the scattering rate of 3.5 meV, is distinctly stronger than that of the theoretical peak-like feature around 369.8 meV in the $\sigma_1^{\mathrm{T}}(\omega)$ of PM $Co_3Sn_2S_2$, which mainly comes from the optical transitions between Band 2 and Band 1 (see the red arrow in Supplementary Fig. 1a) and was calculated with the scattering rate of 50 meV (in the inset of Supplementary Fig. 1c, the theoretical peak-like feature around 369.8 meV in the $\sigma_1^{\mathrm{T}}(\omega)$ of PM $Co_3Sn_2S_2$ was calculated with the scattering rate of 5 meV). Supplementary Fig. 1d shows that the experimental peak-like feature around 217.4 meV in the $\sigma_1^{\mathrm{E}}(\omega,\ T = 8\ \mathrm{K})$ of FM $Co_3Sn_2S_2$ has a stronger intensity than that around 247.8 meV in $\sigma_1^{\mathrm{E}}(\omega,\ T = 200\ \mathrm{K})$ of PM $Co_3Sn_2S_2$, which is consistent with the larger intensity of the theoretical peak-like feature around 319.7 meV arising from the optical transitions between Subband 2↑ and Subband 1↑. Therefore, the sharp decrease in the intensities of the theoretical and experimental optical-conductivity peaks in the energy range from 150 meV to 400 meV can be regarded as spectroscopic evidence for the absence of the splitting of Band 1 and Band 2 into the four subbands: Subband 1↑, Subband 1↓, Subband 2↑ and Subband 2↓ in the PM state of $Co_3Sn_2S_2$. It is worth noticing that in the FM state of $Co_3Sn_2S_2$, the crossing of Subband 2↑ and Subband 1↑, which will not be broken in three pairs of discrete points of the first Brillouin zone, leads to the presence of Weyl points and the formation of Weyl cones. Thus, the absence of Subband 2↑ and Subband 1↑ in its PM state means the disappearance of Weyl semimetal phase in this system. The sharp decrease in the intensities of the theoretical and experimental optical-conductivity peaks in the energy range from 150 meV to 500 meV, which means that the optical transitions are not associated with the two spin-polarized subbands: Subband 2↑ and Subband 1↑, but come from the two spin-degenerate bands: Band 1 and Band 2, therefore supports the absence of Weyl semimetal phase in $Co_3Sn_2S_2$ in its PM state.

Supplementary Note 2. Theoretical Drude weights obtained with the different scattering rates

The scattering rate of the Drude part of the optical conductivity cannot be determined by our single-particle *ab initio* calculations. Generally, the cutoff frequency ω_{c}, which was chosen as the energy position of the minimum of the calculated optical conductivity below the interband transition, will change with the scattering rate Γ used in our single-particle *ab initio* calculations. In order to justify that the choice of the cutoff energy $\omega_{\mathrm{c}} = 38.9$ meV in our manuscript will not influence the theoretical Drude spectral weight of ferromagnetic $Co_3Sn_2S_2$, we further calculated the real parts $\sigma_1^{\mathrm{T}}(\omega)$ of its optical conductivity using the four scattering rates (i.e., Γ = 5 meV, 10 meV,

20 meV and 40 meV) which are different form the scattering rate $\Gamma = 3.5$ meV in our manuscript. Supplementary Fig. 4a shows the theoretical $\sigma_1^{\mathrm{T}}(\omega)$ of ferromagnetic $Co_3Sn_2S_2$ calculated using the scattering rates $\Gamma = 3.5$ meV in the manuscript, 5 meV, 10 meV, 20 meV and 40 meV (To better display the calculated Drude parts, we plotted the low-energy parts of the theoretical $\sigma_1^{\mathrm{T}}(\omega)$ in Supplementary Fig. 4b). As the scattering rates increase, the corresponding cutoff energies, which are indicated by the colored triangles in Supplementary Fig. 4a here, become larger, i.e., $\omega_{\mathrm{c}} = 38.9$ meV in the manuscript, 43.2 meV, 54.2 meV, 80.3 meV and 101.6 meV. Then, integrating the four theoretical $\sigma_1^{\mathrm{T}}(\omega)$ up to the cutoff energies $\omega_{\mathrm{c}} = 43.2$ meV, 54.2 meV, 80.3 meV and 101.6 meV yields approximately the spectral weights: $S^{\mathrm{T}}(\omega_{\mathrm{c}} = 43.2\ \mathrm{meV}) \approx 1.8 \times 10^6\ \Omega^{-1}\ \mathrm{cm}^{-2}$, $S^{\mathrm{T}}(\omega_{\mathrm{c}} = 54.2\ \mathrm{meV}) \approx 1.8 \times 10^6\ \Omega^{-1}\ \mathrm{cm}^{-2}$, $S^{\mathrm{T}}(\omega_{\mathrm{c}} = 80.3\ \mathrm{meV}) \approx 1.8 \times 10^6\ \Omega^{-1}\ \mathrm{cm}^{-2}$, and $S^{\mathrm{T}}(\omega_{\mathrm{c}} = 101.6\ \mathrm{meV}) \approx 1.8 \times 10^6\ \Omega^{-1}\ \mathrm{cm}^{-2}$, which are consistent with the theoretical spectral weight $S^{\mathrm{T}} \approx 1.8 \times 10^6\ \Omega^{-1}\ \mathrm{cm}^{-2}$ obtained by integrating the theoretical $\sigma_1^{\mathrm{T}}(\omega)$ up to $\omega_{\mathrm{c}} = 38.9$ meV in the manuscript. Therefore, the theoretical spectral weight of the Drude part of the optical conductivity obtained by our single-particle ab initio calculations is not impacted by the choice of the cutoff energy.

Supplementary Note 3. Electronic band structures obtained by HSE06 hybrid functional method and mBJ method

The electronic band structures obtained by HSE06 hybrid functional method and mBJ method are shown in Supplementary Fig. 8a and Supplementary Fig. 8b here, respectively. On the contrary to the case that the band inversion occurs along the momentum direction U-L-Γ (please see the inversion between the band 1 and the band 2 in Supplementary Fig. 8c here), a band inversion does not exist between the band 1 and the band 2 in Supplementary Fig. 8a and Supplementary Fig. 8b here. Previous theoretical investigations have demonstrated that the inversion between the band 1 and the band 2 is essential to the emergence of the Weyl semimetal phase in ferromagnetic $Co_3Sn_2S_2$ (please see the related papers: (1) Xu, Q. *et al. Phys. Rev. B* **97**, 235416 (2018); and (2) Liu, E. *et al. Nat. Phys.* **14**, 1125 (2018)). Therefore, the absence of the inversion between the band 1 and the band 2 in Supplementary Fig. 8a and Supplementary Fig. 8b indicates that the HSE06 hybrid functional method and mBJ method, which are different from the method of single-particle *ab initio* calculations in the main text, cannot correctly capture the electronic band structure of ferromagnetic $Co_3Sn_2S_2$ exhibiting a Weyl semimetal state.

Supplementary Note 4. GGA+U Band structure

Supplementary Fig. 9 shows the band structures of ferromagnetic $Co_3Sn_2S_2$, which were obtained by generalized gradient approximation (GGA) + U calculations without and with spin-orbit coupling. Here, we used the simplified approach introduced by Dudarev *et al.* [1] and an effective $U_{\mathrm{eff}} = U - J = 3.2$ eV in our GGA+U calculations. Around the L point of the Brillouin zone, compared with the inverted band structure derived by our *ab initio* calculations (see the red dashed curves in Fig. 3a of the main text), the bands with $3d$ orbital characters have been pushed away from Fermi level and a bandgap of ~ 0.2 eV opens, so the *Weyl points* in the bulk state, which should be present in the six points of the nodal rings, are expected to be *absent* in the band structures obtained by GGA+U calculations.

Supplementary Note 5. Correlated topological electronic structures

Based on the quasiparticle (QP) Hamiltonian in Equation (4) of Methods section, we find that there are six band crossing points in the first Brillouin Zone (BZ) (see Supplementary Fig. 10(a)) at $E - E_{\mathrm{F}} = 86$ meV, which are same as the DFT results (see Supplementary Fig. 10(b)) at $E - E_{\mathrm{F}} = 57$ meV. In order to identify the Weyl points, we have calculated the evolution of Wannier charge center (WCC) [2, 3] on a sphere that enclosing W_1 and W_2 (see Supplementary Fig. 11).

We depicted the (001) surface spectra in Supplementary Fig. 10(c) based on the QP Hamiltonian by using the iterative Green's function method [4] as implemented in the WannierTools package[5],

and compared with the DFT results in Supplementary Fig. 10(d). We also presented the Fermi surface on the (001) surface cutting on the energy of Weyl points by DMFT (see Supplementary Fig. 10(a)) and by DFT (see Supplementary Fig. 10(b)) . The Fermi arc connects one pair of Weyl point with chirality +1 and -1.

Supplementary Note 6. Additional remarks

Numpy [6] and Matplotlib [7] software package were used to create some of the illustrations.

---

[1] Dudarev, S. L., Botton, G. A., Savrasov, S. Y., Humphreys, C. J. and Sutton, A. P. Electron-energy-loss spectra and the structural stability of nickel oxide: An LSDA+U study. *Phys. Rev. B*, **57**, 1505 (1998).

[2] Soluyanov, A. A. & Vanderbilt, D. Computing topological invariants without inversion symmetry. *Phys. Rev. B* **83,** 235401 (2011).

[3] Weng, H., Fang, C., Fang, Z., Bernevig, B. A. & Dai, X. Weyl semimetal phase in noncentrosymmetric transition-metal monophosphides. *Phys. Rev. X* **5,** 011029 (2015).

[4] Sancho, L. M. P., Sancho, J. M. L. & Rubio, J. Highly convergent schemes for the calculation of bulk and surface Green functions. *Journal of Physics F: Metal Physics.* **15,** 851 (1985).

[5] Wu, Q. S., Zhang, S. N., Song, H. F., Troyer, M. & Soluyanov, A. A. Novel topological materials, topological number, surface state, tight-binding model. *Computer Physics Communications* **224,** 405 (2018).

[6] van der Walt, S., Colbert, S. C. & Varoquaux, G. The NumPy array: a structure for efficient numerical computation. *Computing In Science & Engineering.* **13**, 22 (2011).

[7] Hunter, J. D. Matplotlib: A 2D graphics environment. *Computing In Science & Engineering.* **9**, 90 (2007).
